# Proceedings of a Convention of Professional Dentists

**Published:** 1844-12

**Authors:** 


### ARTICLE IV.

*Proceedings of a Convention of Professional Dentists.* Held August 13th and 14th, 1844, in Cincinnati.

IN compliance with a call from the Cincinnati Association of Surgeon Dentists, in connection wiih other members of the profession in the west, a convention of professional dentists met in Cincinnati, on Tuesday, the 13th of August, 1844, at 11 o'clock, A. M., in the lecture room of the Medical College of Ohio.

Dr. James Taylor of Cincinnati, announced the object of the meeting, by reading the circular of invitation.

On motion of Dr. M. Rogers, Dr. Joseph Taylor of Maysville, Ky., was called to the chair, and William Ross of Newport, Ky., was chosen Secretary.

The following gentlemen were present, viz.

JOSEPH TAYLOR, *Maysville, Ky.;* J. P. ULLERY, *Lawrenceburg, Ia.;* A. D. BIGELOW, *Newark, Ohio;* JAMES CLARK, *Lebanon, Ohio;* D. P. HUNT, *Indianapolis, Ia.;* G. D. TETER, *Ripley, Ohio;* D. B. WHEELER, *Xenia, Ohio;* WM. B. ROSS, *Newport, Ky.;* F. E. SUIRE, *Madison, Ia.;* J. W. COOK, P. KNOWLTON, *Cincinnati, Ohio;* A. BERRY, *Raymond, Miss.;* J. B. ROSS, *Philadelphia, Pa.;* M. ROGERS, JOHN ALLEN, H. CRANE, WM. M. HUNTER, W. J. MADEIRA, CHARLES BONSALL, JAMES TAYLOR, *Cincinnati, Ohio.*

The following gentlemen were present by proxy:

H. THOMPSON, *Columbus, Ohio;* W. BASHAW, *Dayton, Ohio;* B. STRICKLAND, *Cleveland, Ohio;* S. P. HULLIHEN, *Wheeling, Va.;* W. H. GODDARD, *Louisville, Ky.;* WM. R. WINTON, *Dayton, Ia.;* B. B. BROWN, *St. Louis, Mo.,* W. E. IDE, *Zanesville, Ohio.*

On motion of Dr. M. Rogers,

*Resolved*, That it is now expedient to form a society for the promotion of dental science.

On motion of Dr. J. Allen,

*Resolved*, That a committee of five be appointed to draft a constitution for the organization of such a society.

Dr. James Taylor, moved that said committee be appointed by nomination; whereupon, the following gentlemen were chosen, viz. Drs. Allen, Cook, Rogers, Bigelow and Hunt.

On motion, a committee of three were appointed by the chair, as a committee of arrangements. Drs. Teter, Bonsall and Crane, were appointed that committee.

The Convention adjourned, to meet at 3 o'clock, P. M. to hear an address from Dr. Rogers.

*Afternoon Session, 3 o'clock, P. M.*

Convention met pursuant to adjournment, when, according to previous arrangement, Dr. Rogers delivered the opening address.

On motion, the address of Dr. Rogers was received, to be placed on file with the proceedings of the convention.

The minutes of the morning session were read and approved.

On motion of Dr. Taylor, the convention requested the members present to communicate any thing of importance that may have occurred in their practice; also to offer remarks upon the address which had been read.

Several communications were then made, relating principally to the malpractice of filling teeth with mineral paste.

Adjourned to meet at 8 o'clock, P. M.

*Night Session, 8 o'clock, P. M.*

The convention was called to order by the chairman.

The committee on the constitution presented their report.

The following preamble and constitution were then adopted:

PREAMBLE.

The undersigned, practical dentists of the Mississippi Valley, deem it expedient to form an association, for purposes of mutual improvement in the science and practice of our profession.

Desirous of promoting the exercise of that gentlemanly courtesy which should characterize members of liberal professions in both social and professional intercourse; believing, also, that by frequent interchange of opinions and observations in practice, by reporting from time to time, cases of interest as they occur in individual practice, we may do much to elevate the character and standing of our profession, and make it worthy the confidence of an enlightened public. For the accomplishment of these objects, we adopt the following

CONSTITUTION.

ART. I.—This society shall be called the Mississippi Valley Association of Dental Surgeons.

ART. II. Sec. 1.—The officers of this society shall consist of a President, three Vice-Presidents, a Recording, and a Corresponding Secretary, Examining Committee, and Treasurer.

Sec. 2.—The officers shall be elected by ballot at each annual meeting and their duties shall be such as appropriately belong to such officers in other societies.

ART. III.—The society now forming, shall be chosen from members of this convention by ballot, two-thirds of all the votes given being necessary to an election.

ART. IV.—After the formation of this society, all applicants for membership shall be examined by the examining committee, and in order to be received as members, they shall sustain a satisfactory examination on all the branches of dental science; shall be twenty-one years of age, and give sufficient evidence of good moral character.

Those who have diplomas from a respectable dental association, may be received without examination by the committee.

ART. V.—All candidates for membership, recommended by the examining committee, shall be ballotted for; two-thirds of all the votes given, shall be necessary to membership.

ART. VI.—Any member may be expelled from the society for immoral conduct, malpractice or other sufficient cause, on motion of an investigating committee appointed for that purpose, by a vote of two-thirds of all the members present.

ART. VII.—This society shall be composed of two classes, viz. acting and honorary members.

ART. VIII.—Each member at his initiation, shall pay into the treasury of the society, the sum of five dollars, and annually the sum of three dollars.

ART. IX.—The meetings of this society shall be held annually, by adjournment from time to time, and from place to place, agreeably to the will of the society.

ART. X.—The society may receive contributions in money, books or other property, which may be either used or sold in aid of its purposes.

ART. XI.—Seven acting members beside the President or chairman, shall be necessary for the transaction of any business, at the opening of any annual meeting.

ART. XII.—In the event that a quorum cannot be obtained at any regular meeting of the society, the time and place on the following year shall be the same as the preceding year. The President shall have the power, in case of a failure of an annual meeting, to call a meeting of the society at any time and place that may seem proper.

ART. XIII.—This constitution may be amended by a vote of two-thirds of the members present at any annual meeting of the society.

On motion,

*Resolved,* That the convention now go into the election of members of the Mississippi Valley Association of Dental Surgeons.

Drs. Rogers and Taylor were appointed tellers; whereupon, the following persons were duly elected, viz.

JOSEPH TAYLOR, *Maysville, Ky.;* J. P. ULLERY, *Lawrenceburg, Ia.;* A. D. BIGELOW, *Newark, Ohio;* JAMES CLARK, *Lebanon, Ohio;* D. P. HUNT, *Indianapolis, Ia.;* D. B. WHEELER, *Xenia, Ohio;* WM. B. ROSS, *Newport, Ky.;* F. E. SUIRE, *Madison, Ia.;* J. W. COOK and P. KNOWLTON, *Cincinnati, Ohio;* A. BERRY, *Raymond, Miss.;* J. B. ROSS, *Philadelphia, Pa.;* M. ROGERS, JOHN ALLEN, H. CRANE, WM. M. HUNTER, W. J. MADEIRA, CHARLES BONSALL and JAMES TAYLOR, *Cincinnati, Ohio.*

The following gentlemen, present by proxy, were duly elected:

H. THOMPSON, *Columbus, Ohio;* W. BASHAW, *Dayton, Ohio;* B. STRICKLAND, *Cleveland, Ohio;* S. P. HULLIHEN, *Wheeling,*

*Va.;* W. H. GODDARD, *Louisville, Ky.;* WM. R. WINTON, *Dayton, Ia.;* B. B. BROWN, *St. Louis, Mo.;* W. E. IDE, *Zanesville, Ohio.*

Adjourned, to meet to-morrow morning at 8 o'clock.

*Wednesday Morning*, 8 *o'clock.*

Convention met, and was called to order by the chairman. Minutes read and approved.

The following officers were then elected for the ensuing year, viz.

| | | |
|---|---|---|
| PRESIDENT, | JESSE W. COOK, *Cincinnati, Ohio.* | |
| 1st V. PR'S, | A. D. BIGELOW, *Newark, Ohio.* | |
| 2d " " | JOSEPH TAYLOR, *Maysville, Ky.* | |
| 3d " " | D. P. HUNT, *Indianapolis, Ia.* | |
| REC. SEC., | W. B. ROSS, *Newport, Ky.* | |
| COR. " | JAMES TAYLOR, *Cincinnati, Ohio.* | |
| EX. COM. | M. ROGERS, " " | |
| | JOHN ALLEN, " " | |
| | F. E. SUIRE, *Madison, Ia.* | |
| TREAS'R, | CHARLES BONSALL, *Cincinnati, Ohio.* | |

The convention having terminated its business by the election of officers, for the Mississippi Valley Association of Dental Surgeons, the society was organized by the President elect, Dr. Jesse W. Cook, being conducted to the chair by the presiding officer of the convention, and Dr. W. B. Ross assuming his duties as Secretary.

On motion of Dr. Bonsall, a committee of five was appointed by the chair to draft by-laws for the government of the society, to report at the next meeting. M. Rogers, James Taylor, A. Berry, P. Knowlton and John Allen, were appointed said committee.

After which an address was read before the society by Dr. James Taylor, upon medico-dental education.

On motion the address was accepted and ordered to be filed among the archives of the society.

On motion Dr. Wm. B. Diver of Cincinnati, was elected an honorary member of this society.

On motion, a vote of thanks was tendered the officers of the convention for the faithful and impartial performance of their duties.

Adjourned to meet at 8 o'clock, P. M.

*Night Session, 8 o'clock.*

The society met, and was called to order by the chairman.

The minutes were read, amended, and approved.

The committee on by-laws, presented their report, which was accepted, and with slight alterations adopted, as follows:

BY-LAWS.

Art. I. Sec. 1.—It shall be the duty of the President to preside at all meetings of the society, to confer all honorary distinctions awarded by the society, whether medals or other testimonials and to draw on the Treasurer for all moneys appropriated by the society.

Sec. 2.—It shall be the duty of the Vice-President, (according to their precedence) to preside at all meetings of the society in the absence of the President.

Sec. 3.—A President pro tem. shall be appointed at any meeting of the society in the absence of the President and Vice-Presidents.

Sec. 4.—It shall be the duty of the Recording Secretary, to keep a record of all the proceedings of the society, file all its books and papers, and to countersign all orders drawn on the Treasurer by the President.

Sec. 5.—It shall be the duty of the Corresponding Secretary, to conduct all correspondence of the society as expressed by the president of the society, or emergency may require.

Sec. 6.—It shall be the duty of the Treasurer to keep all the moneys of the society, to pay out to the order of the President, countersigned by the Recording Secretary, and to keep a correct account of the same.

Sec. 7.—There shall be a publishing committee appointed, whose duty it shall be to superintend the printing and publishing of all books, tracts and other documents the society shall direct.

Art. II.—Honorary members shall be entitled to a seat at all meetings of the society, and shall have the privilege of participating in all debates not involving pecuniary expenditure.

Art. III.—It shall be the duty of the society, at each annual meeting, to appoint members to prepare dissertations on some subject connected with the profession, and also one member to deliver an opening address at its next meeting.

Art. IV.—This society may award premiums to members for dissertations on subjects that may at any time be specified by the society at an annual meeting; also for important improvements in mechanical dentistry, the manufacture of incorruptible teeth or any other suitable object.

Art. V. Sec. 1.—No member shall be permitted to speak more than twice on any question, without express permission of the society, and then only fifteen minutes at each time.

Sec. 2.—Any member shall be liable to be called to order for personal recrimination, or wandering in his remarks from the subject of debate.

Art. VI. Sec. 1.—No member of this society shall take a student for a less time than two years, unless he shall have studied dentistry with some other reputable practitioner, so as to make his term of pupilage equal to two years. It is provided, however, that a graduate of any regular medical college, may be received for the term of one year.

Sec. 2.—Any member violating the preceding section, shall be liable to impeachment and expulsion from this society.

Art. VII.—These by-laws may be altered or amended at any annual meeting of this society, by a vote of two-thirds of the members present.

On motion, Professor M. B. Wright of the Medical College of Ohio, Professor L. M. Lawson of the Transylvania University, and professor C. A. Harris of the Dental College of Baltimore, were elected honorary members of this society.

On motion,

*Resolved,* That the society now suspend its proceedings to hear an address from Dr. J. Allen of Cincinnati.

Dr. Allen then read an address upon the history of dental science, which was, on motion, accepted, to be placed on file among the archives of the society.

On motion, Drs. Cook, Rogers, and James Taylor, were elected a publishing committee.

On motion, Dr. Cook was appointed to deliver the opening address at the next annual meeting:

On motion, Dr. Harris of Baltimore, Dr. Brown of St. Louis, Dr. Ide of Zanesville, Drs. Taylor and Rogers of Cincinnati, and Dr. Goddard of Louisville, were appointed to deliver addresses at the next annual meeting of this society.

On motion,

*Resolved*, That a committee of three be appointed as a board of censors, to decide on the publication of the papers read to the convention and society. J. W. Cook, Charles Bonsall, W. B. Ross, were appointed that committee.

The committee of arrangements made a report, which was accepted.

On motion of Dr. Cook,

*Resolved*, That the Corresponding Secretary be, and he is hereby instructed to give public notice, at least two months prior to the next meeting of the dental society now formed, in the newspapers having the greatest circulation in the west, and in the Western Lancet and Dental Journal.

On motion of Dr. James Taylor,

*Resolved*, That we consider the use of all mineral or other paste in the plugging of teeth, as unprofessional and highly injurious, and that we will neither use it nor countenance its use by others.

*Resolved*, That we present to the board of trustees, and the faculty of the Medical College of Ohio, our sincere thanks for their very kind civility in tendering the use of their spacious and commodious rooms, for the accommodation of this convention.

On motion of Dr. Allen,

*Resolved*, That we now adjourn, to meet in the Medical College of Ohio, on the third Tuesday in August, 1845, at 11 o'clock, A. M.

JESSE W. COOK, *President*.

Wm. B. Ross, *Secretary*.

